# Botulism: Its Nature, Symptoms and Treatment

**Published:** 1918-05-04

**Authors:** Reginald C. Jewesbury

**Affiliations:** Assistant Physician, Charing Cross Hospital.


### BOTULISM.

### ITS NATURE, SYMPTOMS AND TREATMENT.

By REGINALD C. JEWESBURY, M.D., F.R.C.P., Assistant Physician, Charing Cross Hospital.

CONSIDERABLE interest has been aroused recently by the occurrence of a so-called "new" disease known as "botulism." In Great Britain this is, fortunately, very rare, but it has been well known in Germany for several years. The disease is caused by the *Bacillus botulinus*, which gets its name from the Latin word *botulus*, a sausage, because the organism has been found to be present from time to time in sausages. The bacillus was first isolated and described by Van Ermengen in 1896, and belongs to the class of anaerobes, *i.e.* it grows without air. It has been found in ham, bacon, pork, tinned meats and fish, and even butcher's meat, but sausages and tinned foods seem particularly liable to become infected by it. The organism itself is readily destroyed by raising it to a high temperature (60° C. or more), but unfortunately it possesses the power of forming spores and a poison or toxin, which is not so easily rendered harmless. In this property it closely resembles the organisms of diphtheria and tetanus, which also are capable of forming dangerous "toxins."

### SYMPTOMS.

After eating food contaminated with the *Bacillus botulinus* symptoms are liable to develop within a period of from twelve to twenty-four hours (period of incubation). The mouth becomes dry, owing to arrest of the secretion of the salivary glands. The toxin acts specially on the important nerve centres at the base of the brain, causing paralysis of the eye muscles, which manifests itself in dilatation of the pupils, squint, inability to raise the eyelids (ptosis), and nystagmus or oscillating movements of the eyeballs. In addition, there may be difficulty in swallowing and loss of voice. The bowels are constipated and the urine is retained. There may or may not be vomiting. The disease produces a condition of marked general weakness, without any actual paralysis of the limbs. The mind usually remains clear, apart from a condition of general apathy and lethargy, but sometimes there is delirium resembling that of delirium tremens. The face assumes a curious expressionless, masklike appearance. Pain is not a marked feature. Skin rashes are present, though not always. As a rule the disease is unaccompanied by fever, but a temperature of 100 to 101 is not infrequent. In fatal cases death is caused by the poison acting on the nerve-centres which preside over respiration and the heart.

### TREATMENT.

The cases vary in severity, but those which recover extend over a period of several weeks. No change has been found in the blood or cerebro-spinal fluid. In short, the toxin formed by and liberated by the organism causes an acute bulbar paralysis. *Post mortem* but little is found except that the mucous membrane of the pharynx is white and dry, having a "parchment-like" appearance. The spleen is congested, and somewhat enlarged. Signs of inflammation are present in the large bowel. The disease is a serious one, but by no means hopeless. The treatment consists in clearing out the bowel with purges, such as calomel, and giving strychnine at intervals to stimulate the vital centres of the brain which are affected. The most reasonable form of treatment would be in the administration of an anti-toxic serum. Such a serum has been produced by Kempner, but none is available at present. It is to be hoped that a new supply will soon be forthcoming.

### CAUSES AND THE MORAL.

Several cases of this disease have occurred during the past few weeks in different parts of London and in the provinces, and the question is asked, Why? There appears to be no doubt that it is a food poison, and in these times, when our food supply has undergone considerable modifications, it is of the utmost importance to try to trace this very unpleasant malady to some particular class of food. Large quantities of tinned foods have been, and are still, being consumed daily without ill-effects, and it is impossible to condemn these as a whole. It must be remembered that it is particularly raw or partially cooked foods which are found to be dangerous, and that is why the disease is so very much more prevalent in Germany, where it is the custom to eat raw ham, etc. It is not easy to detect infected food, since it does not acquire any tainted odour as a rule, but is said in some cases to have a faintly rancid smell. In addition to meats and fish, the organism has sometimes been found present in cheese. The condition is not one which need cause any general alarm. So far the cases have been comparatively few, and it has not occurred in epidemic form. At the same time, it is one which the general public should be fully alive to, and the means of prevention must be thoroughly understood. The moral is to cook thoroughly all meat, all fish—in particular sausages—pig-meat, and all tinned preparations. If this is done, the chances of becoming a victim of botulism are very remote.

